# Light-inducible T cell engagers trigger, tune, and shape the activation of primary T cells

**DOI:** 10.1073/pnas.2302500120

**Published:** 2023-09-18

**Authors:** Morgane Jaeger, Amandine Anastasio, Léa Chamy, Sophie Brustlein, Renaud Vincentelli, Fabien Durbesson, Julien Gigan, Morgane Thépaut, Rémy Char, Maud Boussand, Mathias Lechelon, Rafael J. Argüello, Didier Marguet, Hai-Tao He, Rémi Lasserre

**Affiliations:** ^a^Aix Marseille Université, Centre National de la Recherche Scientifique, Institut National de la Santé et de la Recherche Médicale, Centre d’Immunologie de Marseille Luminy, Turing Center for Living Systems, 13 288 Marseille, France; ^b^Aix Marseille Université, Institut National de la Santé et de la Recherche Médicale, Institut de neurobiologie de la Méditerranée, Turing Center for Living Systems, 13 273 Marseille, France; ^c^Aix Marseille Université, Centre National de la Recherche Scientifique, Architecture et Fonction des Macromolécules Biologiques, 13 288 Marseille, France

**Keywords:** T cell activation, immunology, optogenetics, TCR, bispecific T cell engagers

## Abstract

To mount appropriate responses, T cells integrate complex sequences of receptor stimuli perceived during transient interactions with antigen-presenting cells. Although it has been hypothesized that the dynamics of these interactions influence the outcome of T cell activation, methodological limitations have hindered its formal demonstration. Here, we have engineered the Light-inducible T cell engager (LiTE) system, a recombinant optogenetics-based molecular tool targeting the T cell receptor (TCR). The LiTE system constitutes a reversible molecular switch displaying exquisite reactivity. As proof of concept, we dissect how specific temporal patterns of TCR stimulation shape T cell activation. We established that CD4^+^ T cells respond to intermittent TCR stimulation more efficiently than their CD8^+^ T cells counterparts and provide evidence that distinct sequences of TCR stimulation encode different cytokine programs. Finally, we show that the LiTE system could be exploited to create light-activated bispecific T cell engagers and manipulate tumor cell killing. Overall, the LiTE system provides opportunities to understand how T cells integrate TCR stimulations and to trigger T cell cytotoxicity with high spatiotemporal control.

## Significance

T cells have an exquisite ability to sense environmental cues to mount an appropriate immune response, but how the dynamic parameters of TCR (T cell receptor) stimulation influence the activation outcome remains poorly understood. Here, we have developed the Light-inducible T cell Engager (LiTE), an optogenetic-based recombinant extracellular TCR agonist that allows the reversible control of TCR stimulation with light on intact primary murine T cells. This technology has allowed us to evidence that the temporal pattern of TCR stimulation directly influences the cytokine program of the T cells. In addition, we have adapted the LiTE system to generate a bispecific T cell engager that enables light-controlled tumor cell killing in vitro.

In addition to the nature and intensity of a stimulus, its dynamic features (frequency, duration) also constitute a layer of information that is decoded by cells and translated into specific outcomes (1). In the lymph node, T cells are highly motile and their activation is usually initiated through transient and sequential interactions with different antigen-presenting cells (APCs) presenting the cognate antigenic peptide bound to major histocompatibility molecule (pMHC) (2–5). At each interaction, the signaling of the T cell receptor (TCR) is triggered (6) and T cells appear able to integrate, or sum, the signals perceived during a series of TCR stimulations (7–10). The temporal pattern of TCR stimulation is thought to influence the outcome of T cell activation (3, 11), although no causal relation has yet been formally demonstrated. A lack of methods enabling an accurate and reversible spatiotemporal manipulation of primary T cell stimulation has, to date, hindered our understanding of the cellular mechanisms behind the integration of activation signals and of the influence of signal dynamics on the activation outcome.

Optogenetics is a technology based on the use of light-responsive protein domains from plants, algae, or prokaryotes to confer light-responsive capacity to targeted biological processes (12). The spatiotemporal control of cellular activities permitted with light has presented new opportunities in fundamental and biomedical research. Recently, optogenetic systems have been implemented in T cell models such as T cell lines or transduced preactivated T cells, including chimeric antigen receptor T cells (CAR T cells) (9, 13–17). These elegant methodologies offered a new capacity to manipulate T cells and provided insight into the mechanism of T cell activation. However, they rely on the transduction into cells of genes encoding modified light-responsive proteins, preventing to study primary T cells that are known to be resistant to genetic modifications without previous in vitro preactivation.

In the present study, we have developed the LiTE system, an original optogenetic recombinant extracellular tool to control in space and time TCR stimulation using light. We demonstrated that the LiTE system delivers accurate, tunable, and reversible TCR stimulation and can efficiently induce T cell effector functions. This molecular system showed exquisite reactivity to photostimulations, is fully reversible, and displayed remarkable robustness as it keeps its functionality over days. To highlight the potential of the LiTE system, we deciphered T cell response to intermittent TCR stimulation, whose dynamic parameters mimic the duration and frequency of T cell interactions with APCs during their priming in vivo. It revealed that CD4^+^ T cells are more prone to respond to intermittent stimulations than CD8^+^ T cells. Moreover, we have reported evidence that distinct sequences of TCR stimulation encode different cytokine programs, demonstrating a causal relationship linking TCR stimulation dynamics and the outcome of T cell activation. Furthermore, we have extended the LiTE system concept to generate the LiTE-Me system, a light-inducible bispecific T cell engager that enables light-targeted melanoma cell killing, in vitro. Therefore, by providing original control on untouched primary T cell stimulation, the LiTE system offers opportunities to study and manipulate these cells.

## Results

### Design and Validation of the LiTE System, An Optogenetics-Based Recombinant Molecular System for Reversible Control of TCR Stimulations with Light.

**Fig. 1.**
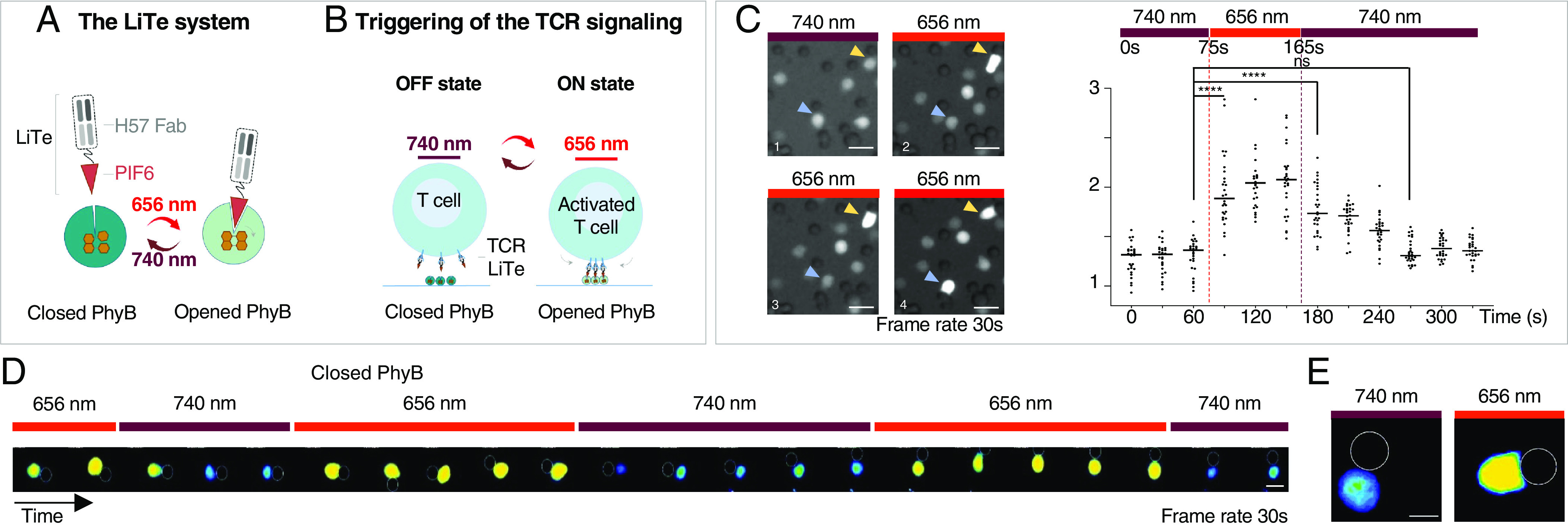
Design of the LiTE system for reversible triggering of TCR signaling in response to light. (*A*) The LiTE system is in two parts: LiTE protein, a recombinant protein linking the PIF6 domain to the anti-mouse TCR H57-597 Fab and PhyB_1-651_. Exposure of the LiTE system to red light opens the PhyB binding site for PIF6, whereas far-red light reverses this conformational change. (*B*) Exposure of mouse T cells in the presence of the LiTE system to 656 nm light induces the binding of TCR-bound LiTE proteins to PhyB-coated surface, leading to TCR aggregation/immobilization and triggering. This process is reversed by 730- to 760 nm light. (*C*) Primary T lymphocytes were loaded with PBX, a calcium-sensitive dye, then incubated with the LiTE protein before being mixed with PhyB-coated beads and imaged by fluorescence microscopy at 37 °C. (*Left*) Time-lapse images of the cells exposed to 740 nm light and then to 656 nm light in the presence of the LiTE system. Arrows indicate cells in contact with PhyB-coated beads over time. (*Right*) Quantification of relative fluorescence intensity of cells in contact with PhyB-coated beads and exposed to the specified light sources (n = 30 cells, representative of >3 experiments; ****P < 0.0001, Wilcoxon–Mann–Whitney test; (scale bar: 10 μm). (*D*) Time-lapse images of a T cell under iterative stimulation/resting cycles (scale bar: 5 μm, frame rate: 30 s). (*E*) Morphological changes of a lymphocyte in contact with PhyB-coated bead and in response to exposure to 740 nm (*Left*) or 656 nm (*Right*) lights; (scale bar: 5 μm.)

We engineered the LiTE system, a recombinant and reversible light-inducible stimulatory system to control the activation of untouched primary T cells. Its rationale relies on the fact that a nonagonist monovalent Fab can become a potent agonist upon oligomerization (18). The LiTE system has two components: i) the N-terminal phytochrome B_1-651_ region from *Arabidopsis thaliana* linked to the phycocyanobilin (PhyB) and ii) the LiTE protein that combines the phytochrome interacting factor 6 (PIF6) to the Fab fragment from the H57 mAb, an agonist targeting the mouse TCR β-chain (Fig. 1*A* and *SI Appendix*, Fig. S1). Under red light illumination (e.g., 630 to 656 nm), PhyB captures the PIF6 domain of the LiTE protein. Far-red illumination (e.g., 740 to 780 nm) abrogates this interaction. Once the LiTE proteins bind TCRs, their light-controlled capture by PhyB-coated supports induces reversible TCR triggering (Fig. 1*B*). We validated the ability of the LiTE protein to bind specifically to the TCR (*SI Appendix*, Fig. S2 *A* and *B*). Next, we verified that its interaction with PhyB occurred only under red light exposure (*SI Appendix*, Fig. S2 *C* and *D*).

We evaluated the performance of the LiTE system on living primary T cells by recording its ability to trigger intracellular calcium influx as an early, sensitive, and dynamic readout of TCR engagement (10, 19, 20). Primary CD8^+^ T cells were loaded with PBX calcium-sensitive dye and the LiTE protein before incubation with PhyB-coated beads; they were then imaged with a videomicroscope at 37 °C. Switching light from 740 nm to 656 nm triggered an increase in intracellular calcium within tens of seconds solely for T cells in contact with PhyB-coated beads (Fig. 1*C* and *SI Appendix*, Fig. S3). The calcium concentration gradually returned to the basal level within 2 min after switching the light back to 740 nm. These dynamics are consistent with previous observations for TCR-induced calcium influx during antigen recognition engagement (10, 19, 20). Moreover, the LiTE system enabled the delivery of pulsed stimuli to T cells via the application of iterative cycles of 656/740 nm light exposure, as shown by the synchronization of intracellular calcium fluxes with the light cycle dynamics (Fig. 1*D*). Also, under 656 nm light, the shape of T cells in contact with PhyB-coated beads was reminiscent of that reported at the immunological synapse; this suggests the engagement of a large number of TCRs (Fig. 1*E*). Altogether, these observations demonstrate that the LiTE system has the unique ability to operate on primary T cells without requiring their genetic manipulation. It constitutes an ON/OFF molecular switch enabling precise spatiotemporal control of T cell activation via strong, reversible, and iterative TCR stimulation.

**Fig. 2.**
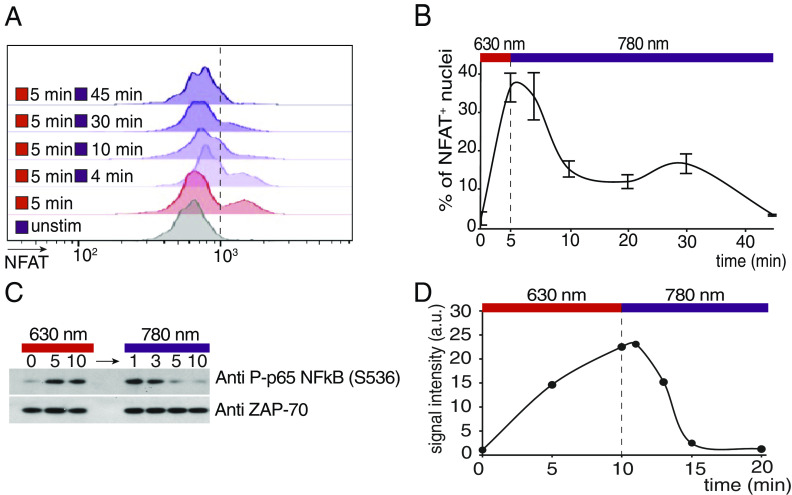
Kinetics of NFAT nuclear translocation and NFκB phosphorylation in response to transient LiTE-driven TCR stimulation. Primary CD8^+^ T cells were incubated with the LiTE system and illuminated on optoPlate with 630 nm light for the indicated time and then with 780 nm light for the indicated time. (*A*) Flow cytometry analysis of the amount of NFAT in purified T cell nuclei at different time points (representative of n = 2 experiments). (*B*) Percentage of NFAT positive nuclei shown in *A*. (*C*) Western blot analysis of NFκB phosphorylation during illumination at a wavelength of 630 nm followed by illumination at 780 nm (representative of n = 2 experiments). (*D*) Quantification of NFκB phosphorylation signal shown in *C*. Values are normalized on the ZAP-70 molecule signal as loading control.

To further verify the ability of the LiTE system to stimulate TCR signaling pathways, we evaluated its capability to trigger downstream signaling events such as transcription factor activation. We first analyzed the nuclear translocation of Nuclear Factor of activated T-cells (NFAT), a key transcription factor activated by the calcium-dependent signaling pathway. Primary CD8^+^ T cells incubated with the LiTE system were stimulated with 630 nm light illumination for 5 min; then, stimulation was interrupted by switching the light to 780 nm. At different time points, T cell nuclei were isolated to assess NFAT translocation by flow cytometry (Fig. 2 *A* and *B*). These experiments showed that, while NFAT translocates rapidly into the nucleus after LiTE-induced stimulation, it exhibits a slow export when stimulation is interrupted. In fact, NFAT returned to its basal nuclear concentration only 45 min after the interruption of the stimulation. These observations show that the LiTE system provides an efficient TCR stimulation that mobilizes transcription factors. In addition, it shows that while calcium influx ceases rapidly upon cessation of TCR stimulation, the downstream nuclear translocation of NFAT follows different kinetics, being more persistent.

We also investigated the dynamics of induction of the NFκB axis, another key transcription factor for T cell activation. T cells were stimulated with the LiTE system; then, the stimulation was interrupted and the phosphorylation of NFκB was analyzed at different time points by western blot (Fig. 2 *C* and *D*). This experiment showed that NFκB is rapidly phosphorylated after TCR stimulation and rapidly dephosphorylated after cessation of the stimulation. In fact, it returns to its basal level of phosphorylation within 5 min. Therefore, NFκB follows a distinct activation/deactivation kinetics than NFAT during transient TCR stimulation. Overall, the LiTE-driven TCR stimulation induces a signal transduction cascade leading to transcription factor activation.

### The LiTE System Triggers T cell Effector Functions and Lays the Foundation for Light-Inducible Bispecific T cell Engagers for Tumor Cell Killing.

**Fig. 3.**
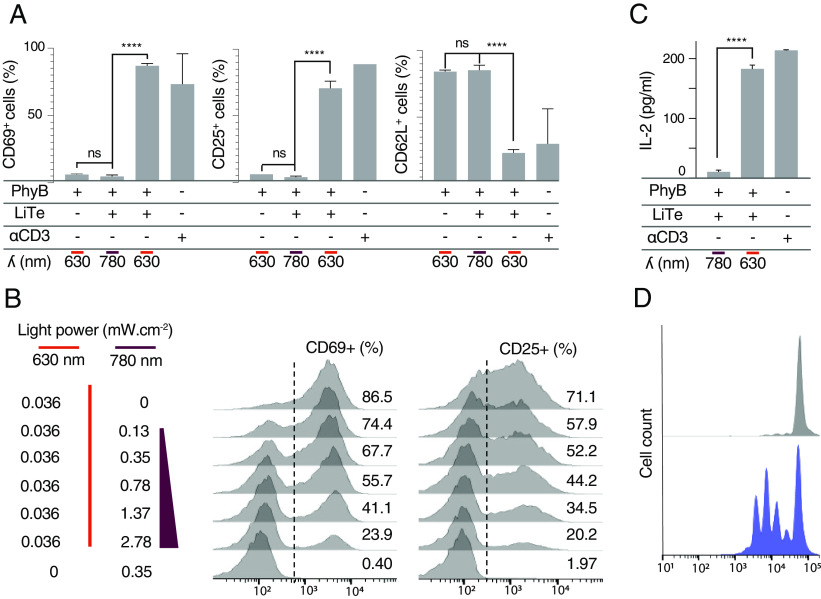
LiTE-driven TCR photostimulation is tunable and leads to effective T cell activation. (*A*) Primary T cells were incubated with the LiTE system and anti-CD28 antibody, then illuminated 18 h in optoPlate at the specified wavelength. Flow cytometry analysis of CD69, CD25, or CD62L positive cells in response to 630- or 780 nm light (n > 3, mean ± SEM are shown; ***P < 0.001, ****P < 0.0001, Student *t* test). (*B*) Same experimental conditions as in *A*, except that cells were exposed to different ratios of 630 nm/780 nm light power and analyzed for the expression of CD69 or CD25 by flow cytometry. (*C*) Same experimental conditions as in *A*, except that IL-2 secretion was analyzed in the cell supernatant by ELISA (n = 3; mean ± SD are shown; ****P < 0.0001, Student *t* test). (*D*) T Cells treated as in a were illuminated 72 h with 780 nm light (*Upper*) or 630 nm light (*Lower*) before the analysis of their proliferation by measurement of CellTrace Violet dilution by flow cytometry (representative of n > 3 experiments).

We next wanted to determine whether the LiTE system could induce functional activation of primary T cells. Activated T cells are characterized by the surface expression of activation markers as well as by the onset of effector functions such as cytokine secretion or cytotoxicity. Thus, primary CD8^+^ T cells were incubated with the LiTE system, in the presence of soluble anti-CD28 mAbs that increased the fraction of responding cells (*SI Appendix*, Fig. S4). Cells were placed on an illumination device [optoPlate-96, (21)] and illuminated for 18 h with light at 630 nm (ON), or 780 nm (OFF). Next, T cell activation was assessed by flow cytometric analysis of activation markers (Fig. 3*A*). Under 780 nm light conditions, the cells remained in the resting state. In contrast, the 630 nm light triggered potent T cell activation, as observed by an increase in CD69 and CD25 expression, and a decrease in CD62-L. Under these conditions, up to 80% of the T cells were activated such as with anti-CD3/anti-CD28 stimulation. As expected, neither the 630 nm light nor LiTE system alone could induce any T cell activation (*SI Appendix*, Fig. S5*A*). Also, no phototoxicity was noted with the low light power used in these experiments (0.14 mW cm^−2^ at 630 nm and 2.8 mW cm^−2^ at 780 nm; *SI Appendix*, Fig. S5*B*). We then determined whether the LiTE system permits the tuning of T cell activation using combined illuminations at different ratios of 630 nm to 780 nm light power (Fig. 3*B*). Flow cytometry analysis of CD69 and CD25 up-regulation revealed a proportional relationship between T cell activation and the ratio of 630 nm to 780 nm light power. Therefore, the LiTE system allowed adjustable control of the extent of T cell activation.

To assess the ability of the LiTE system to induce T cell effector functions, we first monitored cytokine production. Purified primary CD8^+^ T cells were stimulated as above with 630 nm light, and secreted IL-2 was quantified by enzyme-linked immunosorbent assay (ELISA) (Fig. 3*C*). The LiTE system appeared very effective at inducing IL-2 production by the T cells, only when exposed to 630 nm light. Next, to further characterize the LiTE-induced activation, the proliferation of T cells treated as above and exposed to 630 nm or 780 nm light for 72 h was analyzed by flow cytometry (Fig. 3*D*). We observed that the LiTE system induces a strong proliferation of the T cells only under 630 nm light exposure. Overall, this demonstrates the ability of the LiTE system to induce full T cell activation. It also reveals that this recombinant molecular system is capable of delivering long-term stimulations.

**Fig. 4.**
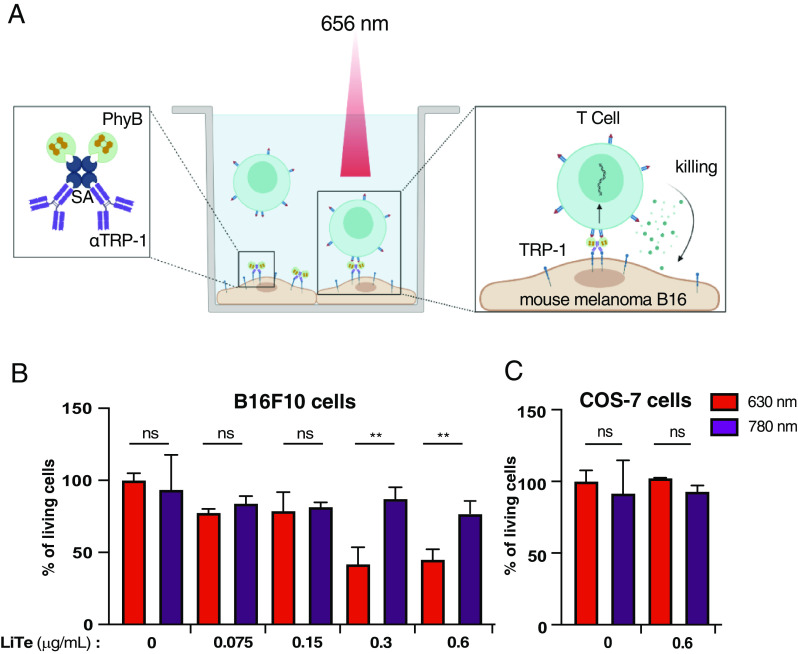
LiTE-Me system design and validation. (*A*) PhyB is combined with streptavidin (SA) and with the melanoma cell-targeting antibody TA-99 (specific for surface molecule TRP-1). When bound to melanoma cells, the Me–PhyB complex can engage LiTE-bound CTL when exposed to 656 nm light and trigger tumor cell killing. (*B*) CTLs were dropped on a B16F10 melanoma cell monolayer in the presence of the LiTE-Me system at the indicated concentration and then illuminated with a 630 nm or 780 nm light for 18 h at 37 °C. Tumor cell killing has been evaluated with the CellTiter-Glo® luminescent assay; the histogram represents the percent of living tumor cells compared to untreated cells (i.e., without the LiTE-Me system), (n = 2; mean ± SD are shown; **P < 0.001, Student *t* test). (*C*) Control under the same conditions as in A but with the TRP-1 negative COS-7 cell line; (n = 2; mean ± SD are shown, Student *t* test).

A fundamental effector function of the CD8^+^ T cell compartment is the killing of pathological cells, such as cancer cells. We therefore wished to evaluate the capacity of the LiTE system to trigger tumor cell killing by syngenic CD8^+^ effector T cells. To this end, we modified the LiTE system to generate the LiTE-Me system in which PhyB is targeted to the surface of melanoma cells by coupling to a mAb specific for Tyrosinase-related protein 1 (TRP-1), a melanoma-associated antigen (Fig. 4*A*). In practice, we generated and purified molecular complexes containing streptavidin and biotinylated forms of PhyB and anti-TRP1 mAb (Fig. 4*A* and *SI Appendix*, Fig. S6 *A* and *B*). We verified that these molecular complexes were able to bind specifically to TRP-1 expressing B16F10 melanoma cells (*SI Appendix*, Fig. S6*C*). Then, CD8^+^ effector T cells with different concentrations of the LiTE-Me system were dropped on B16F10 melanoma cells and illuminated with 630 nm or 780 nm light for 18 h. The cell Titre Glo luminescent viability assay was used to examine the viability of B16F10 cells. This study showed that nearly 60% of tumor cells were killed by cytotoxic T cells, but only when the cells were in the presence of the LiTE-Me system at a sufficient concentration (above 0.3 µg/mL) and illuminated by a 630 nm light (Fig. 4*B*). In addition, no LiTE-Me-induced tumor cell killing was observed with COS-7 cells, which do not express TRP-1 (Fig. 4*C*). Thus, the LiTE-Me system enables specific killing of tumor cells by cytotoxic T cells under the control of light.

Altogether, these experiments show that the LiTE system enables specific and adjustable activation of primary T cells, leading to the establishment of their effector functions. The LiTE-Me system provides control of T cell activity at the cell/cell interface, without the use of beads or any artificial surfaces. More importantly, we provide in vitro evidence that LiTE-Me acts as a light-inducible bispecific T cell engager (BiTE), allowing spatiotemporal control with light of tumor cell killing by CD8^+^ effector T cells.

### The Temporal Pattern of TCR Stimulation Shapes the Activation Outcome of the T cell Compartment.

Previous studies have suggested that the dynamics of T cell/APC interactions influences the outcome of T cell activation (3, 11). Yet, the causal relationship has not been formally demonstrated. With this in mind, we next wished to exploit the flexibility of the LiTE system to explore how the temporal pattern of the TCR stimulation influences the outcome of T cell population activation. We also aimed to determine whether the LiTE system could allow manipulation/orientation of the adaptive immune response.

**Fig. 5.**
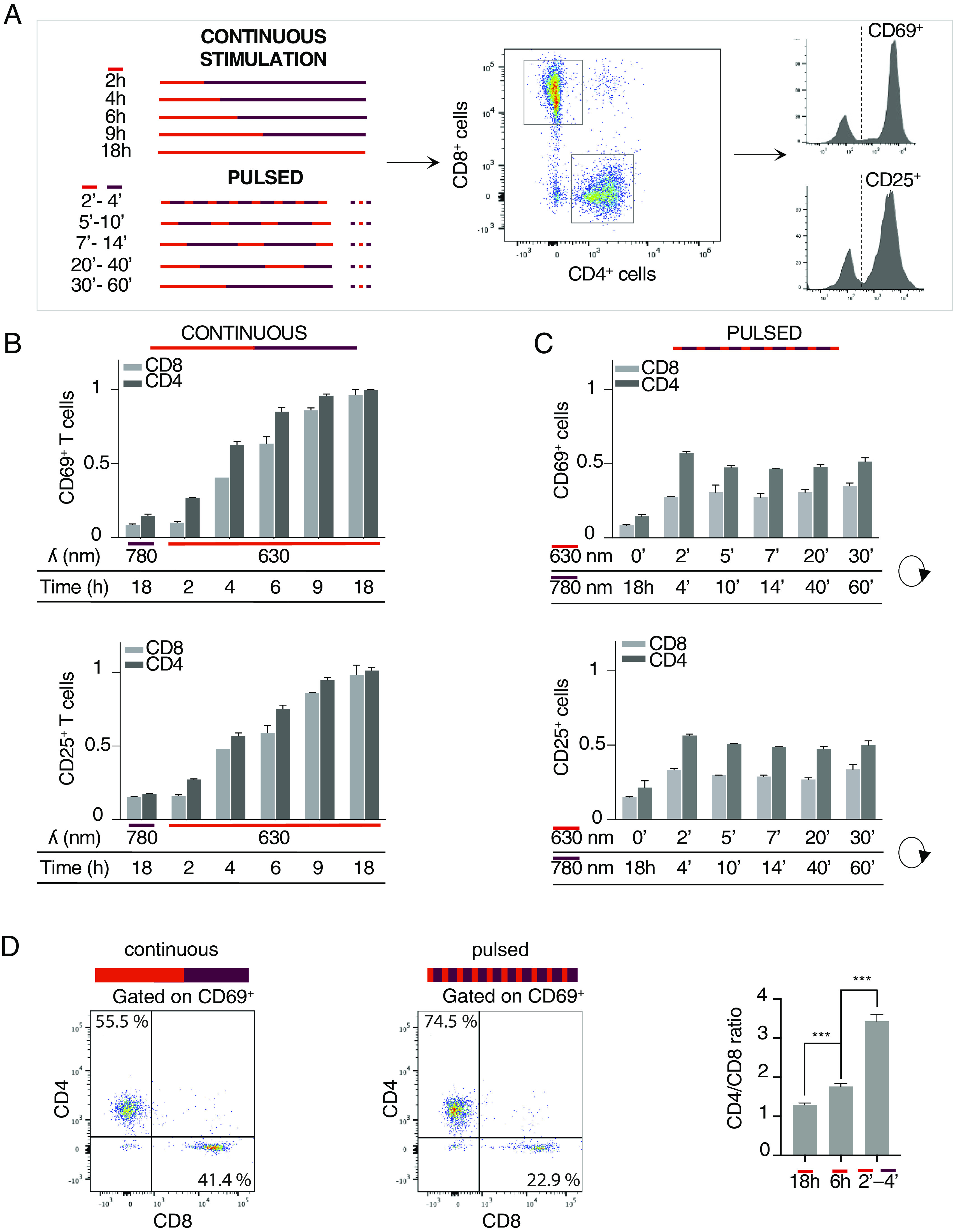
LiTE system enabled the differential mobilization of the CD4^+^ and CD8^+^ T cell subsets. (*A*) Primary T cells were incubated with the LiTE system and anti-CD28 antibody and then illuminated for 18 h in optoPlate under continuous or pulsed illumination conditions. All pulsed illumination conditions correspond to 6 h of cumulative exposure time to 630 nm light. CD4^+^ and CD8^+^ T cell subsets were analyzed by flow cytometry for CD69 and CD25 expression. (*B*) Flow cytometry analysis of the T cell activation following different periods of continuous photostimulation (n > 3; mean ± SEM are shown; normalized data to 18-h continuous photostimulation value). (*C*) Same as in *B*, but for pulsed photostimulation of different durations (n > 3; mean ± SEM; normalized data to 18 h continuous photostimulation value). (*D*) Dot plot showing the proportion of CD4^+^ and CD8^+^ T cells among the CD69^+^ T cells, activated under continuous (*Left*) or pulsed photostimulation conditions (*Middle*). A total of 2,500 activated cells are shown in each panel. The CD4^+^/CD8^+^ T cell ratios induced by the indicated stimulation conditions are shown in the *Right* panel. The 2′-4′ pulsed stimulation condition over 18 h corresponds to a cumulated 6-h exposure to the 630 nm light (representative of n > 3 experiments; mean ± SD are shown; *** indicates *P* < 0.0005, Student *t* test).

We first confirmed that PhyB remained functional after hours of exposure to illumination cycles alternating between 630 nm and 780 nm light (*SI Appendix*, Fig. S7). Then, purified primary T cells were incubated with the LiTE system under different conditions of light exposure and soluble anti-CD28 mAb. Cells were stimulated with time-gated 630 nm light exposure in continuous or pulsed mode, and then, the CD69 and CD25 up-regulation in CD8^+^ T and CD4^+^ T cell subsets was analyzed by flow cytometry (Fig. 5*A*). Upon continuous TCR stimulations, the fraction of cells up-regulating CD69 and CD25 increased with the duration of 630 nm light exposure, to similar extents for the CD8^+^ and CD4^+^ subsets although the latter responded slightly better to short stimulations (Fig. 5*B*). However, when T cells were exposed to different temporal patterns of pulsed stimulation for 18 h, CD4^+^ T cells clearly showed a higher response than CD8^+^ T cells (Fig. 5*C*). For the short 2-min ON/4-min OFF cycles, the proportion of activated CD4^+^ T cells was up to threefold higher than the proportion of CD8^+^ T cells (74.5% vs. 22.9% of the CD69^+^ cells, respectively, Fig. 5 *D*, *Left*). In comparison to continuous stimulations, pulsed stimulations favored the activation of CD4^+^ T cells over CD8^+^ T cells, as illustrated by the ratios of activated CD4^+^/CD8^+^ T cells (Fig. 5 *D*, *Right*). Of note, the duty cycles were held constant (33%, 6 h of cumulated ON state over an 18-h period) between different pulse cycles that ranged from 6 to 90 min. The cycle length had only a moderate influence on primary T cell activation (Fig. 5*C*).

**Fig. 6.**
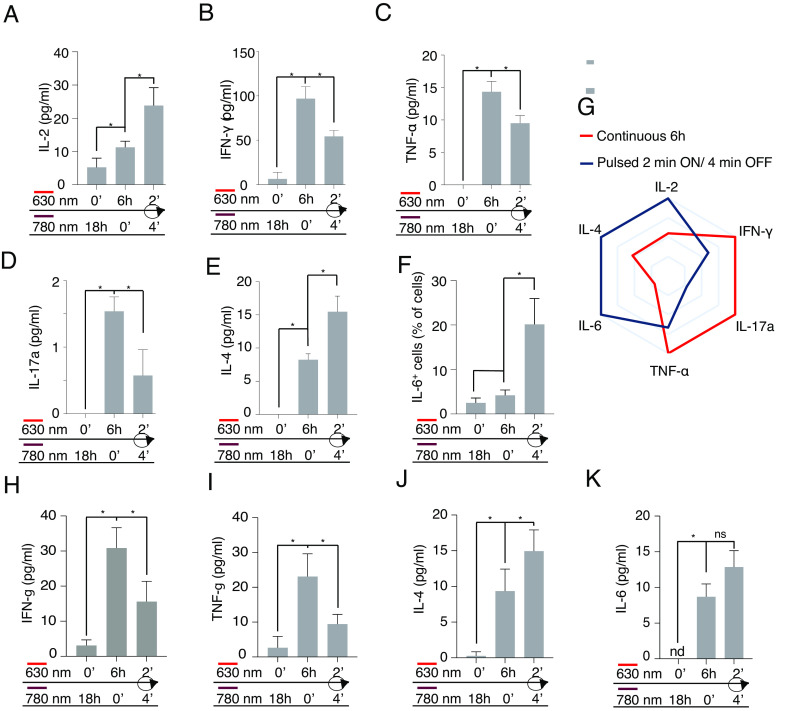
Distinct sequences of TCR stimulation encode different cytokine programs. Primary T cells were incubated with the LiTE system and anti-CD28 antibody and then illuminated for 18 h in optoPlate under continuous or pulsed illumination conditions (6 h of cumulated stimulation time). (*A*) Measurement of IL-2 secretion in the supernatants by ELISA. (*B*–*E*) Measurement by the multiplex assay of IFN-γ, TNF-α IL-17, or IL-4 secretion, respectively. (*F*) Analysis of intracellular IL-6 by flow cytometry (n = 2; mean ± SD; *P < 0.05, Wilcoxon–Mann–Whitney test). (*G*) Polar representation of the cytokine panels produced by the T lymphocytes stimulated after continuous (in blue) or pulsed (in red) stimulations shown in *A*–*F* (normalized to the maximum value detected for each cytokine). (*H*–*K*) Measurement by the multiplex assay of the quantity of IFN-γ, TNF-α, IL-4, and IL-6, respectively, secreted by primary naive CD4^+^ T cells stimulated as in *A*, (n = 2; mean ± SD; *P < 0.05, Wilcoxon–Mann–Whitney test).

The differential responses shown by T cell subsets to pulsed stimulations raised the possibility of using the LiTE system to qualitatively manipulate the activation of the T cell compartment. We therefore characterized the panel of cytokines produced by primary T cells following 6-h continuous or pulsed stimulations (iterative 2-min ON/4-min OFF during 18h). Interestingly, pulsed stimulations of T cells induced higher amounts of production IL-2, IL-4, and IL-6 than continuous stimulations (Fig. 6 *A*, *E*, and *F*). On the contrary, T cells stimulated with pulsed stimulations showed a decreased production of IFN-γ, TNF-α and IL-17, compared to T cells under continuous TCR stimulations (Fig. 6 *B*–*D*). Therefore, the dynamics of TCR stimulation influenced the panel of cytokines secreted by the T cell population (Fig. 6*G*). The differences observed in the production of IL-4 and IL-6, two cytokines mainly produced by CD4^+^ T cells, suggested that the dynamics of TCR stimulation influenced not only the balance of the CD4^+^ T cell/CD8^+^ T cell response but also the nature of the CD4^+^ T cell response. To test this hypothesis, naive CD4^+^ T cells were isolated, stimulated with the LiTE system under the same conditions, and the cytokines produced were analyzed by the multiplex assay (Fig. 6 *H*–*K*). We observed that, as for the mixed T cell populations, intermittent TCR stimulation favors the production of IL-4 and, to a lesser exent, IL-6 by the naive CD4^+^ cells, whereas continuous stimulation significantly favors IFN-γ and TNF-α production. These results suggest that continuous TCR stimulation fosters a Th1-like response, illustrated by higher levels of IFN-γ and TNF-α production, whereas intermittent TCR stimulation likely promotes a Th2-like response associated with increased production of IL-4.

Altogether, these experiments revealed a remarkably enhanced ability of CD4^+^ T cells to integrate a series of short-lived TCR stimulations, when compared to CD8^+^ T cells. They also demonstrated that distinct sequences of TCR stimulation encode different cytokine programs. Finally, the combination of the LiTE system with specific illumination strategies allows therefore the manipulation of T cell compartment activation and the release of associated cytokines.

## Discussion

Optogenetics is a promising technology for manipulating cellular activity in fundamental and biomedical research. However, it has been limited, to some degree, by the constraint of genetically modifying the target cells, making difficult the study or manipulation of primary cells. Our results in primary T cells show that use of the fully recombinant LiTE system can overcome this limitation. By way of its modular design, the LiTE system concept can be easily adapted to other biological systems.

By providing the spatiotemporal control of TCR stimulation, the LiTE system enables both quantitative and qualitative manipulation of T cell activation. As mentioned above, nearly two decades ago, it was proposed that the dynamics of T cell stimulation could qualitatively influence the cellular response (3, 11). We provide here direct evidence of a causal relationship linking the dynamics of TCR stimulation and the outcome of T cell activation. By using our original LiTE technologies, we reported that intermittent TCR stimulation favors Th2 differentiation. This observation is consistent with studies indicating that induction of the Th2 response is associated with T cell/DC interactions of shorter duration than those observed during Th1 response induction (22, 23). It shows that T cells are able to decode the dynamics of stimulation of the TCR to set an appropriate response and place the signal dynamics as a layer of information for the adaptive immune system. In this context, we have observed that when TCR stimulation is interrupted, the transcription factor NFAT can persist in the nucleus for tenths of minutes, where it likely remains active (11), while other transcription factors such as NFκB do not. Such differences in the kinetics of activity of transcription factors downstream of the TCR could allow the T cell to encode different temporal patterns of TCR stimulation into specific intracellular signaling schemes that ultimately drive different cytokine programs. In line with this hypothesis, studies have shown that while NFκB synergizes with NFAT for IFN-γ gene transactivation (24), it has been reported to inhibit NFAT-dependent IL-4 gene transcription (25). Further research is needed to refine our knowledge of this phenomenon and to determine the extent to which controlling the dynamics of TCR stimulation with the LiTE system would enable to influence TCR signaling and to trigger the selective induction of different T cell activation programs (e.g., tolerance, TH-1, TH-2, or TH-17 responses…). In this context, Achar et al have recently reported that distinct cellular responses of the T cells are elicited by antigenic peptides of different affinity for the OT-I TCR (26). Peptide affinity influences both the half-life of TCR-pMHC binding and the stability of T cell/APC interactions (27). As the LiTE system, based on PhyB photoactivation, enables the control of the duration of TCR engagement at the molecular level (13), but also at the cellular level, it could provide opportunities to uncover the molecular mechanisms underlying the induction of such a diversity of cellular responses.

In an elegant study, O’Donoghue et al. have generated human T cells expressing an optogenetic-based light-controllable CAR [OptoCAR, (9)]. By applying intermittent CAR photostimulations, they showed that CAR T cells filter out specific stimulation frequencies, responding drastically less to stimulation cycles with a period around 25 min. Here, if our data show a similar trend in murine primary T cells, variations in response amplitude to stimulation cycles of different periods were less pronounced in our study. This difference could be explained by the biological models used: While O’Donoghue et al. have studied transduced human CAR T cells, we studied murine primary T cells that were not preactivated. Also, TCR/CD3 receptor signaling may significantly differ from the one of CD3ζ-based CAR receptor.

In the lymph node, phase I of T cell priming, characterized by a series of short-lived interactions with APCs, has been reported to be shorter for CD4^+^ T cells than for CD8^+^ T cells (2, 4, 28). However, as CD4^+^ and CD8^+^ T cell dynamics have been studied in different TCR transgenic mouse models, it is unclear whether this difference is related to the biological models or whether it reflects the intrinsic ability of these two T cell subsets to respond to intermittent stimulations. Here, we report that CD4^+^ T cells respond to intermittent TCR stimulation more efficiently than their CD8^+^ T cell counterparts. This was made possible because the LiTE system targets the Cβ domain on TCR, an epitope shared by all αβ T cells. Such an intrinsic difference may explain, at least in part, the earlier up-regulation of CD69 on CD4^+^ T cell subtype during priming.

The ability to selectively stimulate T cell subtypes or manipulate the outcome of T cell activation in vitro could also be highly relevant in the field of cellular immunotherapy. Indeed, the efficacy of cell-based immunotherapies, including in vitro stimulation of tumor-infiltrating lymphocytes or of circulating T cells for CAR T cell generation, is highly dependent on the differentiation state of the T cells as well as on the T cell subtypes expanded (29–31). It would therefore be interesting to exploit the original level of control provided by the LiTE system to optimize the in vitro T cell activation procedures for cell-based immunotherapies.

With new technologies to illuminate optogenetic tools in tissue (32–34), optogenetics is now being applied in oncoimmunology research and has enabled the production of light-inducible CAR T cells in mice (16, 17). In this context, the LiTE-Me system presented here has provided a proof-of-concept of the relevance of optogenetics to also control the bioactivity of BiTEs by light. BiTEs are promising therapies against cancer, but their usage is sometimes limited by the induction of severe adverse effects due to an immune activation at the systemic level, which can be very deleterious and sometimes lethal. Overcoming these adverse effects is a central challenge in oncoimmunology (35). Spatiotemporal control by light of the activity of light-responsive BiTEs, such as the LiTE-Me system, could be a relevant strategy to generate highly targeted immunotherapies with decreased adverse effects. Furthermore, considering the ability of the LiTE system to shape the outcome of T cell activation in response to specific time-gated photostimulations, light-controllable BiTEs could provide a great level of control of the BiTE-induced antitumor immune response. Indeed, differences in the cytokine produced in the tumor bed can have a strong influence on the recruitment and function of bystander T cells, NK cells, macrophages, neutrophils, and other stromal cells. Future studies will be necessary to test LiTE-Me in vivo and determine the specific improvements needed to fully unleash its therapeutic potential. Its regulation with light in the red and the far-red regions, which penetrate tissues at the millimeter scale, constitutes a great advantage for these applications (34).

Overall, the LiTE system constitutes an innovative and versatile molecular tool that allows precise spatiotemporal control of primary cells. It provided us the capacity to directly demonstrate the influence of the TCR stimulation dynamics on the outcome of T cell activation. Moreover, this molecular system opens up broad avenues in biomedical research for the development of future therapeutic approaches.

## Material and Methods

### Reagents and Antibodies.

Dulbecco’s modified Eagle medium (DMEM), Roswell Park Memorial Institute (RPMI) medium, DMEM/Nutrient Mixture F-12 (DMEM/F-12), sodium pyruvate (NaPy), L-glutamine (L-Glu), penicillin-streptomycin (Pen-Strep), geneticin, acide 4-(2-hydroxyéthyl)-1-pipérazine éthane sulfonique (HEPES), and β2-mercaptoethanol were purchased from Gibco. Phosphate-buffered saline (PBS), streptomycin sulfate salt, biotin, tris (2-carboxyethyl) phosphine (TCEP), Tween 20, N-dodecyl β-D-maltoside and valproic acid sodium salt were supplied by Sigma Aldrich. Streptavidin-HRP was purchased from Beckman Coulter. Alexa Fluor 488 and caspase-3/7 activity detection dyes were purchased from Thermo Fisher and Viobility™ 405/452 Fixable Dye from Miltenyi Biotec. The monoclonal antibodies anti-CD28 (clone H37.51) and goat anti-mouse IgG-HRP were purchased from Thermo Fisher, APC anti-CD69 (clone REA937) and FITC anti-CD62L (clone MEL-14) from BioLegend, FITC anti-CD25 (clone 7D4) and anti-6×His (clone F24-796) from BD Biosciences, AF647 anti-histidine (clone AD1.1.10) from Bio-RAD, and APC anti-CD45 (clone 30-F11) from eBioscience™. The AF488 anti-NFAT1 (D43B1) and anti-Phospho-NF-κB p65 (Ser536) (93H1) were purchased from Cell Signaling Technologies. The TA-99 antibody (α-TRP1) was a gift from Innate Pharma SA. The anti-TCR β (clone H57-597) and anti-CD3ε (clone 145-2C11) were purified from hybridoma culture supernatants following standard protocols. H57-597 Fab fragment was prepared by papain digestion, purified by ion exchange, and controlled by sodium dodecyl sulfate-polyacrilamide gel electophoresis (SDS-PAGE). Fluorescent labeling of proteins was performed according to the manufacturer’s instructions.

### Cell Culture.

The melanoma B16F10 cell line was cultured in the RPMI medium supplemented with 10% fetal bovine serum (FBS) with 7.5% CO_2_ at 37 °C. The MCD4 cell line was derived from the mouse 3A9 CD4^+^ T cell hybridoma expressing a high TCR level specific for hen egg lysozyme peptide bound to MHC II I-Ak molecules, as previously described (36). MCD4 cells were grown in RPMI medium supplemented with 5% FBS, 1 mM NaPy, and 10 mM HEPES with 10% CO_2_ at 37 °C. The human embryonic kidney 293T cell line (HEK293T) was grown in DMEM supplemented with 10% FBS, 1 mM NaPy, 2 mM L-Glu, and geneticin with 7.5% CO_2_.

Primary T lymphocytes were isolated from lymph nodes of C57BL/6 mice using the Dynabeads™ Untouched™ mouse T cell kit (Invitrogen™) by negative selection. CD8^+^ T cells were isolated from lymph nodes of C57BL/6 Rag1^−/+^ OT-1^−/+^ mice and purified using the EasySep™ mouse CD8^+^ T cell isolation kit (STEMCELL Technologies) by negative selection. T cells were cultured in complete DMEM/F-12 medium (DMEM/F-12 supplemented with 10% FBS, 1 mM NaPy, 10 mM HEPES, 50 U/mL Pen-Strep, and 0.05 mM β2-mercaptoethanol) with 10% CO_2_ at 37 °C.

The effector CD8^+^ T cells were cultured in 6-well plates at 0.625 × 10^6^ cells/mL in complete DMEM/F-12 medium with 1 μg/mL anti-CD28 (clone H37.51). The 6-well plates were coated with 3 μg/mL anti-CD3ε (145-2C11) in PBS for 4 h at 37 °C and washed three times with PBS before seeding the T cells. Cells were cultured for 48 h (37 °C, 7.5% CO_2_), after which IL-2 (PeproTech) was added to a final concentration of 10 U/mL. The cells were then cultured for a further 48 h.

### Generation and Production of PhyB.

PhyB was produced in BL21(DE3) *Escherichia coli* (Sigma Aldrich) transfected with three plasmids obtained from the laboratory of K. Rosen and Leung (37) that encode the heme oxygenase 1 (HO1), phytochromobilin synthase lacking the transit peptide (ΔHY2), and phytochrome B (PhyB_1-651_). The latter was modified to add a Strep-tag at the C terminus of PhyB_1-651_. Alternatively, we used the plasmid pMH1105 (38), a gift from Dr Wilfried Weber (Addgene, plasmid # 131864; http://n2t.net/addgene:131864; RRID:Addgene_131864) to produce a biotinylated form of PhyB; this plasmid encodes the PhyB_1-651_, HO1, and PCB:ferredoxin oxidoreductase (PcyA). For the production of PhyB, the transfected bacteria were grown at 30 °C in lysogeny broth medium supplemented with the selection antibiotics and then induced with 1 mM Isopropyl-β-D-1-thiogalactopyranoside at OD 600 nm between 0.6 and 0.8 and grown overnight at 18 °C in the dark at 150 rpm. For biotinylated PhyB production, biotin was added (5 µM) to the culture medium allowing the biotinylation of the Avi-Tag. Bacteria were harvested by centrifugation for 8 min at 6,500 g and 4 °C and then resuspended in lysis buffer (50 mM HEPES, 500 mM NaCl, 5% glycerol, 0.5 mM TCEP, and 20 mM imidazole, pH 7.4) and shock frozen in liquid nitrogen before being stored at −80 °C. Bacteria were disrupted by adding DNAse (10 µg/mL) and MgSO_4_ (20 mM) during 20 min and were then sonicated. Lysate was cleared from debris by centrifugation at 30,000 g and 4 °C for 30 min before being loaded onto a Nickel-NTA Superflow cartridge (Qiagen) using an Äkta Explorer chromatography system (GE Healthcare) and then purified. The purified protein buffer was exchanged to PBS containing 0.5 mM TCEP using a HiPrep 26/10 desalting column (GE Healthcare).

### Generation and Production of the LiTE protein.

In order to produce the LiTE protein, gene encoding the H57 Fab Light Chain (LC) was cloned into pTT22 eukaryotic expression vector (pTT22-H57Fab-LC plasmid) and the H57 Fab Heavy Chain (HC) linked to the PIF6 domain with a 6×His tag (*SI Appendix*, Fig. S1) within the pYD7 eukaryotic expression vector (pYD7-H57Fab-HC-PIF plasmid). All cloning reactions were performed using the In-Fusion HD cloning kit (Clontech) in a standard reaction mixture.

The HEK293T cell line was then transfected with both the pYD7-H57Fab-HC-PIF plasmid and the pTT22-H57Fab-LC plasmid (ratio 1:3) using polyethylenimine (Polysciences). Cells were then maintained in DMEM supplemented with 2% FBS, 0.5% Tryptone TN1 (OrganoTechnie), 1.25 mM valproic acid, and geneticin, at 37 °C with 5% CO2. Supernatants were harvested 7 d later, and the protein was purified by Ni-NTA affinity chromatography. The purified protein buffer was exchanged to PBS using a Slide-A-Lyzer™ Dialysis Cassette (Thermo Fisher).

### Western Blot Analysis.

Purified proteins in reduced 5× Laemmli loading buffer were denatured for 5 min at 95 °C before being loaded onto a 10% SDS–PAGE gel, separated by electrophoresis and transferred onto nitrocellulose membrane (LI-COR). Membranes were then blocked with 4% bovine serum albumin in TBS/T buffer (137 mM NaCl, 20 mM Tris pH 7.6, 0.1% Tween 20). Proteins were detected with reagents coupled to HRP and revealed by chemiluminescence with the Pierce™ ECL Western kit (Thermo Scientific™) and Azure biosystems 300 imaging system.

For P-NFκB analysis, T cell were stimulated with the LiTE system following the indicated conditions and then lyzed 10 min in lysis buffer (50 mM Tris pH 7.5, 0.5 mM Ethylenediaminetetraacetic acid (EDTA), 137 mM NaCl, 10% glycerol, and 0.2% N-dodecyl β-D-maltoside). After a 10-min centrifugation at 1,000 g, supernatants were collected and boiled 5 min in the presence of β-mercaptoethanol containing Laemmli buffer before being loaded on a 8% SDS–PAGE gel. Electrophoresis, transfer on membrane, and revelation were processed as described above.

### Flow Cytometry Analysis.

Cells were washed twice with flow cytometry buffer (PBS, 0.5% FBS) and then incubated for 45 min at 10 °C before being labeled with antibodies in flow cytometry buffer. After 3 washes, cells were resuspended in FACS buffer and analyzed by flow cytometry on a Beckton Dickinson (BD) FACSCanto™. Data were analyzed with FlowJo software (Treestar Inc., CA).

For NFAT translocation analysis, T cell nuclei were purified with the Nuclei isolation Kit (NUC201 Sigma-Aldrich), fixed, and permeabilized with the fix/Perm reagents (from 00-5523-00 eBioscience) and then stained with AF488-conjugated anti-NFAT mAb. After 3 washes, nuclei were resuspended in FACS buffer and analyzed by flow cytometry on a BD FACSCanto™. Data were analyzed with FlowJo software (Treestar Inc., CA).

### Spectral Analysis of PhyB Photoswitching.

Spectral analysis of PhyB photoswitching was measured with a NanoDrop™ One (Thermo Scientific™). The absorption spectrum of PhyB was measured after 3 min under light exposure at 680 nm. The samples were then exposed to 740 nm light for 1 h before recording a new absorption spectrum.

### Light-Controlled PhyB/LiTE Pull-Down Assay.

Streptavidin-coated magnetic beads (MagStrep "type3" XT beads 5% suspension, Iba) were washed three times in PBS supplemented with 0.5 mM TCEP with a magnetic separator (Iba). The beads were then incubated with 20 µg/mL of PhyB for 1 h at 4 °C and then washed 3 times before adding the LiTE protein at a concentration of 20 µg/mL. The mix samples were illuminated 10 min with light at 656 nm or 740 nm. After 3 washes, the samples were boiled and the eluted proteins analyzed by western blotting.

### Photostimulations and Calcium Imaging.

A 20 µg/mL solution of PhyB protein was added to washed streptavidin-coated polystyrene beads (5.0 to 5.9 µm, Spherotech), before 2-h incubation on ice. After washing in PBS supplemented with 0.5 mM TCEP, the beads were resuspended in complete DMEM/F-12 medium without phenol-red and distributed in Lab-Tek™ II Chambered Coverglass (Thermo Fisher). Primary T lymphocytes were loaded with PBX calcium reporter according to the manufacturer’s instructions, then washed before incubation with 20 µg/mL of LiTE protein for 45 min at room temperature, and finally washed again before incubation with PhyB-coated beads. The samples were illuminated with a 656 nm or 740 nm LED system (Mightex) and imaged on a videomicroscope, at 37 °C.

### T cell Activation Analyses.

A 20 µg/mL solution of PhyB protein was added to washed streptavidin-coated polystyrene beads and then incubated for 1 h in ice. After washing with PBS supplemented with 0.5 mM TCEP, the beads were resuspended in complete DMEM/F-12 medium. The beads were distributed in black clear-bottom 96-well plates (Greiner). Plates were illuminated on optoPlate for 3 min under 780 nm light to ensure a closed conformation of all PhyB proteins. Primary T cells labeled with 0.3 µg/mL of LiTE protein were added at a concentration of 5 × 10^5^ cells/well in the presence of 2 µg/mL anti-CD28 antibody. The plates were incubated on the optoPlate located in the cell culture incubator and exposed to the indicated photostimulation programs. The harvested T cells were then analyzed by flow cytometry to measure cell surface protein expression and by ELISA of their supernatants to measure IL-2 secretion. Plates were read at 450 nm with a Tecan INFINITE M1000 PRO. For T cell proliferation analysis, T cell were labeled with the CellTrace Violet Cell Proliferation Kit (Thermo Fischer Scientific) for 20 min at 37 °C, before being stimulated. After 72 h of illumination at 630 nm or 780 nm, the CellTrace Violet dilution was analyzed by flow cytometry.

### Melanoma-Targeted PhyB Generation and Enrichment.

Biotinylated PhyB was added to a streptavidin solution in PBS/TCEP with a 2:1 molar ratio under constant vortexing before the addition of an excess of biotinylated TA-99 antibody. Samples were separated by High-performance liquid chromatography (HPLC) with a Superdex 200 10/300-Increase column (Cytiva), and the protein content was evaluated by absorbance at 280 nm and 680 nm for total protein and PhyB detection, respectively. The collected fractions were then analyzed by western blotting and revealed with streptavidin-HRP to detect biotinylated proteins. The complexes from the peak A fraction have been used for the functional assay.

### Measurement of Light-Induced Tumor Cell Killing By Flow Cytometry.

A total of 5 × 10^3^ melanoma B16F10 cells or Cos 7 cells per well were plated in a 96-well plate and loaded with the melanoma-targeted PhyB complex from peak A. Effector CD8^+^ T cells were cocultured at a 5:1 ratio with the target cells in a total volume of 200 µL/well of RPMI supplemented with 10% FBS, 1 mM NaPy, 10 mM HEPES, 0.05 mM β2-mercaptoethanol, and the LiTE protein at the indicated concentration. Cells were illuminated with the indicated light for 18 h at 37 °C. Then, after three washes, the quantity of living tumor cells has been estimated using the CellTiter-Glo® assay (Promega).

### Statistical Analyses.

Quantitative data were reported as means ± SD or ± SEM. Excel (Microsoft) and Prism (GraphPad Software) were used for statistical analyses. The nonparametric Wilcoxon–Mann–Whitney test or the Student *t* test was used for the evaluation of significance, as indicated in the figures. The sample number (*N*) refers to the number of biological replicates. n.s. = not significant (*P* > 0.05), *P < 0.05, **P < 0.01, ***P < 0.001, and ****P < 0.0001.

## Supplementary Material

Appendix 01 (PDF)

## Data Availability

All study data are included in the article and/or *SI Appendix*.
