# Supplementary material for: Light-inducible T cell engagers trigger, tune, and shape the activation of primary T cells
Source: Proc Natl Acad Sci U S A. 2023 Sep 18;120(39):e2302500120. doi: 10.1073/pnas.2302500120 (PMC10523538; doi:10.1073/pnas.2302500120)
Supplement: Supplementary file 1 — Appendix 01 (PDF) [file pnas.2302500120.sapp.pdf]

PNAS

Supporting Information for
Light-inducible T cell engagers trigger, tune and shape the activation of primary T cells

Morgane Jaeger[1], Amandine Anastasio[1], Léa Chamy[1], Sophie Brustlein[2], Renaud Vincentelli[3], Fabien Durbesson[3], Julien Gigean[1,] Morgane Thépaut[1], Rémy Char[1], Maud Boussand[1], Mathias Lechelon[1], Rafael J. Argüello[1], Didier Marguet[1], Hai-Tao He[1] and Rémi Lasserre[1*]

Rémi Lasserre
Email: lasserre@ciml.univ-mrs.fr

This PDF file includes:

Figures S1 to S7
Legends for Datasets S1 to S7

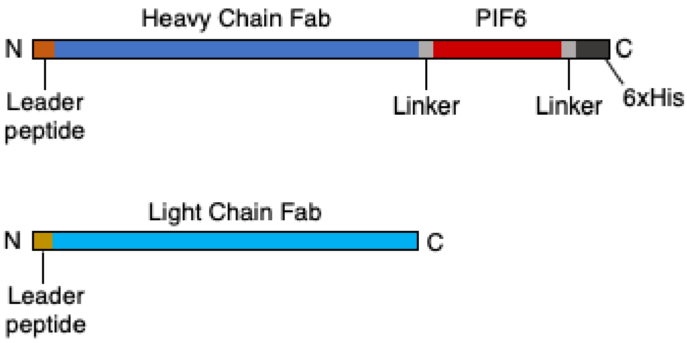

Protein of the Heavy Chain Fab - PIF :
MEFGLSWVFLVALFRGVQCEVYLVESGGDLVQPGSSLKV
SCAASGFTFSDFWMYWVRQAPGKGLEWVGRIKNIPNNY
ATEYADSVRGRFTISRDDSRNSIYLQMNRLRVDDTAIYYC
TRAGRFDHFDYWGQGTMVTVSSASTKGPSVFPLAPSSKS
TSGGTAALGCLVKDYFPEPVTVSWNSGALTSGVHTFPAV
LQSSGLYSLSSVVTVPSSSLGTQTYICNVNHKPSNTKVDK
RVEPKSCDKTGAGSGSGSGSGSMMFLPTDYCCRLSDQE
YMELVFENGQILAKGQRSNVSLHNQRTKSIMDLYEAEYNE
DFMKSIIHGGGGAITNLGDTQVVPQSHVAAAHETNMLESN
KHVDGSGSGSGSGSENLYFQGHHHHHH*

Protein of the Light Chain Fab :
MKYLLPTAAAGLLLLAAQPAMAYELIQPSSASVTVGETVKI
TCSGDQLPKNFAYWFQQKSDKNILLLIYMDNKRPSGIPER
FSGSTSGTTATLTISGAQPEDEAAYYCLSSYGDNNDLVFG
SGTQLTVLRGRTVAAPSVFIFPPSDEQLKSGTASVVCLLN
NFYPREAKVQWKVDNALQSGNSQESVTEQDSKDSTYSL
SSTLTLSKADYEKHKVYACEVTHQGLSSPVTKSFNRGEC*

Fig. S1. Construction of the recombinant LiTE protein. The LiTE protein is produced in transfected eukaryotic cells following transfection of a plasmid encoding the light chain of the H57-597 Fab and the phytochrome interacting factor 6 (PIF6) domain attached by a flexible linker to the C-terminal amino acid sequence of the heavy chain of the H57-597 Fab. A second linker at the C-terminal part of PIF6 comprised the 6xHis tag for the purification and detection of the recombinant LiTE protein.

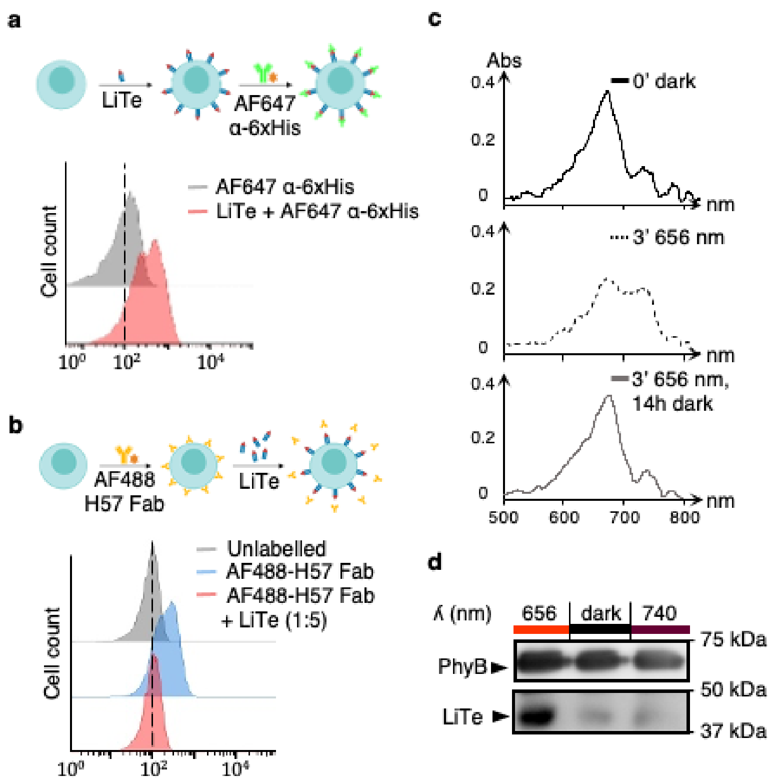

Fig. S2. Evaluation of the functionality of the LiTE system components.

a, Flow cytometry analysis of the binding of LiTE protein to mCD4 T cell line detected by AF647-anti 6xHis mAb. **b**, Displacement of AF488-H57 Fab bound to TCR by the LiTE protein. **c**, Spectral analysis of the PhyB produced in *E. coli*. Top, absorbance of PhyB in a closed conformation, i.e., before 656 nm light illumination. Middle, absorbance spectra in its open conformation induced by a 3 min light exposure at 656 nm. Bottom, same as the middle condition but followed by 14 h incubation in the dark to recover the close conformation of PhyB. **d**, Pull-Down assay of the LiTE protein by PhyB-coated beads co-incubated and exposed to different conditions of illumination (n>3).

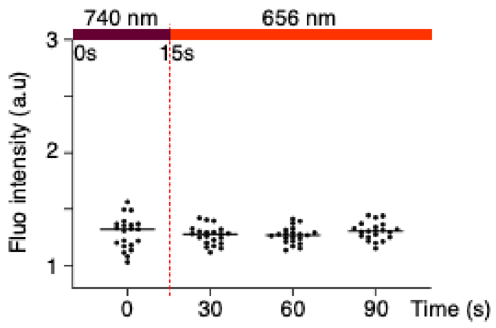

Fig. S3. Light exposure does not induce calcium influx in the absence of PhyB.
Primary T lymphocytes were loaded with the PBX calcium sensitive dye and the LiTE protein, and were then maintained in the absence of PhyB-coated beads. Under these conditions, no calcium influxes were observed under 656 nm light exposure (n=20 cells).

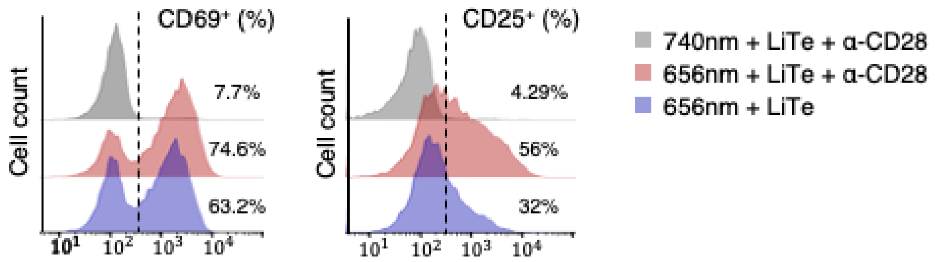

Fig. S4. CD28 costimulation increased the fraction of T cells responding to the LiTE system. Primary CD8 T cells were incubated with the LiTE system in the presence or not of anti-CD28 antibody, then illuminated 12 h in optoPlate at the specified wavelength. Flow cytometry charts showing the T cell surface expression of CD69 (left) and CD25 (right).

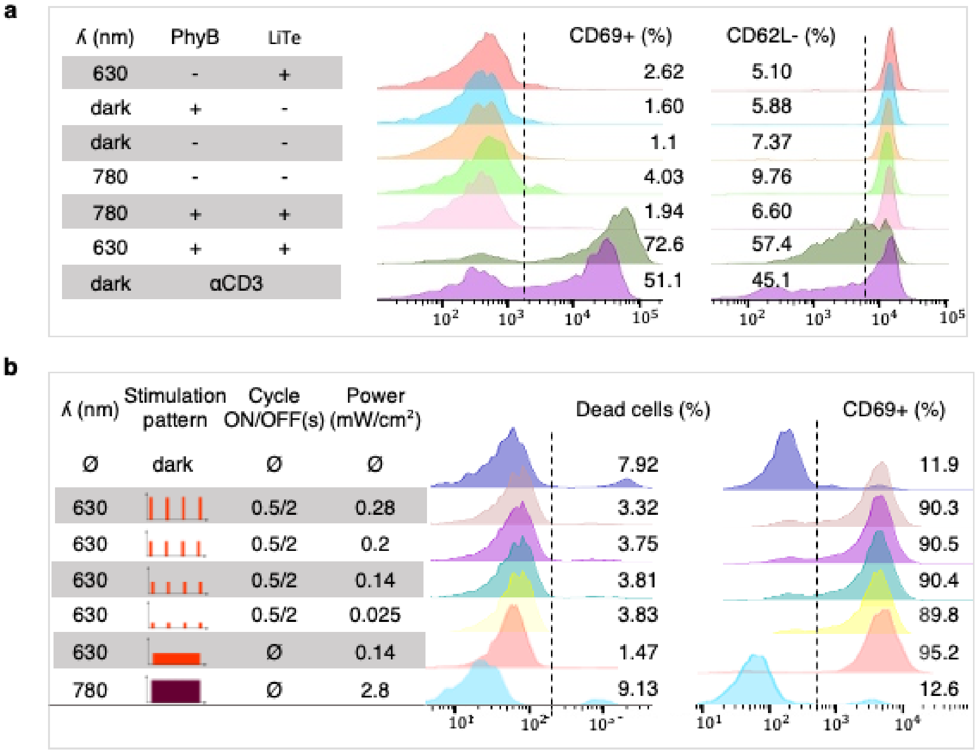

Fig. S5. Control for the LiTE system specificity and for light-induced phototoxicity.

a, Primary T cells were incubated with the LiTE system and anti-CD28 antibody, then illuminated or not for 18 h in the optoPlate at the specified wavelength. Flow cytometry analysis of CD69 and CD62L cell surface expressions in response to 630 or 780 nm light. The activation of T cells required both the LiTE system and red-light exposure. **b**, Same as in **a** but under different illumination conditions. Cell death and T cell activation have been evaluated by flow cytometry using Viobility™ fixable dye and CD69 cell surface expression, respectively. T cell exposure to red or far-red light was not phototoxic.

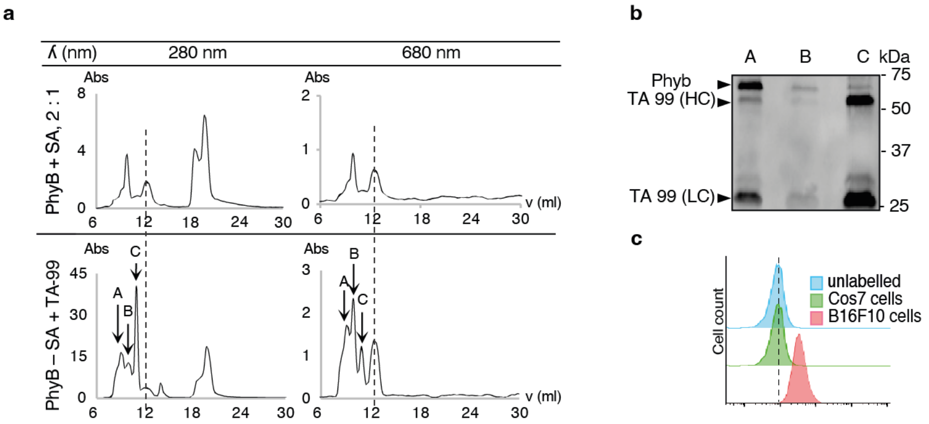

Fig. S6. Strategy for LiTE-Me generation, purification and characterization.

a, Upper panel, complexes of biotinylated PhyB-streptavidin (SA) in 2:1 ratio were analyzed by HPLC followed by absorbance detection at 280 (upper left) and 680 nm (upper right). The dashed line indicates the monomeric form of PhyB. Lower panel, PhyB-SA complexes were incubated with an excess of biotinylated TA-99 mAb and the result analyzed by HPLC followed by absorbance detection at 280 (lower left) and 680 nm (lower right).

The molecular complexes contained in the fraction collected in the peak A have been used for the functional assay. Their average molecular weight was estimated at 417 +/- 40 kDa, based on the Superdex 200 10/300-Increase column calibration. This value is closed to the theorical molecular weight for complexes composed of 1 streptavidin/2 anti-TRP1 mAb/2 PhyB (428 kDa). In addition, this estimation is consistent with the measured ratio between the absorbances at 680 nm and 280nm. While this ratio is 0.35 for purified PhyB, it is approximatively 0.1 in the fraction corresponding to the peak A. It indicates that PhyB constitute close to one third of the total protein content of the peak A fraction (it is the only component that absorbs light at 680 nm). Therefore, the 1 streptavidin/2 anti-TRP1 mAb/2 PhyB complex represents the majority of the molecular species found in this fraction.

b, Western blot analysis of the HPLC fractions collected in A-C peaks shown in **a**. As expected, the A fraction contains high amount of PhyB and of TA-99 mAB.

c, Flow cytometry analysis of the binding of the A peak complex on B16F10 cells, and on COS-7 cells. The complexes from the peak A fraction have been used for the functional assay shown in figure 4.

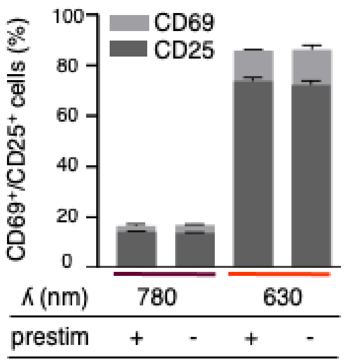

Fig. S7. Conservation of PhyB functionality after iterative 630 nm/780 nm illumination cycles. PhyB-coated beads were exposed for 4 h to 15 min ON/15 min OFF light cycles (prestim) or not. Then, CD8^{+} T cells and LiTE were added and the wells exposed to the indicated illumination for 18 h to evaluate by flow cytometry the percentage of CD69 and CD25 positive cells. (n=2; mean +/- SD are shown).
